# Effect of virtual cement space and restorative materials on the adaptation of CAD-CAM endocrowns

**DOI:** 10.1186/s12903-022-02598-0

**Published:** 2022-12-09

**Authors:** Ziting Zheng, Hebi Wang, Jiayao Mo, Zhiting Ling, Yuting Zeng, Yuxin Zhang, Jilei Wang, Wenjuan Yan

**Affiliations:** 1 Department of Stomatology, Nanfang Hospital, Southern Medical University, 1838 N Guangzhou RD, Guangzhou, Guangdong People’s Republic of China; 2 Department of Stomatology, The Third Affiliated Hospital of Guangzhou University of Chinese Medicine, Guangzhou, Guangdong People’s Republic of China

**Keywords:** Marginal gap, Cement space, Endocrown, Ceramic, Composite resin

## Abstract

**Background:**

This study aimed to evaluate the effect of virtual cement space and restorative materials on the fit of computer-aided design and computer-aided manufacturing (CAD-CAM) endocrowns.

**Methods:**

A mandibular first molar tooth model received a butt joint margin endocrown preparation with a 2-mm occlusal thickness. Then, using a 3D-printing system, 120 copies of this prepared die were printed and assigned equally to three groups with different cement space settings (30, 60, and 120 μm) during the chairside CAD design. In the milling process, CAD-based models with a particular space setting were subdivided into four groups (n = 10) and fabricated from different CAD-CAM materials: Vita Suprinity (VS), Celtra Duo (CD), Lava Ultimate (LU), and Grandio blocs (GR). Finally, the endocrowns were stabilized over their corresponding models with siloxane and subjected to micro-computed tomography to measure the fit.

**Results:**

The cement space that was predesigned at 30 μm generated the largest marginal discrepancy (from 144.68 ± 22.43 μm to 174.36 ± 22.78 μm), which was significantly different from those at 60 μm and 120 μm (*p* < 0.001). The combination of VS or CD with a pre-setting cement space of 60 μm and the combination of LU or GR with a cement space of 120 μm showed better agreement between the predesigned and actual measured marginal gap widths. For internal adaptation, only the cement space set to 30 μm exceeded the clinically acceptable threshold (200 μm).

**Conclusions:**

The setting of the cement space and restorative material significantly affected the marginal adaptation of CAD-CAM endocrown restorations. Considering the discrepancy between design and reality, different virtual cement spaces should be applied to ceramic and resin composite materials.

## Background

Proper fit of crowns and abutments, presented by internal and marginal gaps, affects the long-term success of dental restorations [1]. The internal gap is measured as the perpendicular distance from the internal surface of the restoration to the axial wall of the preparation, and the marginal gap is the perpendicular distance at the cavosurface margin [2]. Poor marginal adaptation causes the dissolution of the cement layer, resulting in secondary caries and periodontal disease, eventually leading to clinical failure [3, 4]. An inappropriate internal gap can increase the thickness of the cement, decrease the adhesive strength at the adhesive interface, and reduce the resistance to fracture of the restoration [1, 5, 6]. Marginal gaps of ≤ 120 μm and internal gaps of ≤ 200 μm have traditionally been considered clinically acceptable [7–9].

Computer-aided design and computer-aided manufacturing (CAD-CAM) dentistry has been widely applied to enhance the effectiveness and accuracy of treatment procedures and outcomes. By chairside designation and production, endocrown restoration has achieved excellent esthetics and favorable biomechanical behavior and has become the preferred choice for many clinicians to restore severely damaged teeth after endodontic treatment [10–12]. In a systematic review, CAD-CAM endocrowns obtained clinical success rates ranging from 94 to 100% [13]. Secondary caries and loss of retention, which are closely related to the dissolution of the luting cement and deficiencies in marginal and internal adaptation, were the main causes of failure in the long-term follow-up of CAD-CAM endocrowns [14]. Therefore, it is essential to better understand the factors affecting the fit of CAD-CAM endocrowns.

The CAD-CAM system setting allows the adjustment of different parameters, including the virtual cement space (CS), during the virtual 3-dimensional (3D) design of the restoration [15]. Setting a certain CS width around the fabricated CAD-CAM restoration is important for proper adaptation between the restoration and the prepared abutments, and for a good distribution of the luting agents [16–19]. Studies have shown that the CS value significantly affects the marginal and internal fit of CAD-CAM crowns [20–22]. Whether CS settings have a similar effect on endocrown restoration requires further investigation. According to previous studies, variations in the setting values of CS around CAD-CAM endocrowns ranging from 40 to 120 μm yield different margins and internal adaptations [4, 6, 7]. Therefore, a detailed study of how the CS setting affects the marginal and internal fit of CAD-CAM endocrowns is important.

In addition to the CS setting, the choice of restorative material also influences the final fit of the restoration. With improvements and innovations in CAD-CAM technology, various materials with different compositions and physical properties have become available as CAD-CAM materials [23, 24]. Ceramic materials are popular because of their high esthetics, biocompatibility, and durability; composite resins possess biomimetic properties close to those of human teeth and are more resistant to higher occlusal forces [7, 25]. However, investigations of the effects of different materials on restoration adaptation have reported conflicting findings. Some studies reported that restorations with ceramic materials provide a more favorable marginal and internal fit than composite resins [7, 26], while others showed opposite results [4, 27–29].

Despite the widespread use of endocrowns, studies focusing on milling parameters for the ideal fit of manufactured pieces are lacking. The choice of setting values for virtual CS and the type of restorative materials remains unclear. Therefore, this study aimed to combine the parameters of virtual CS and restorative materials and evaluate the fit of CAD-CAM endocrown restorations to determine the best combination. The null hypothesis was that neither the CS nor the type of restorative material would influence the marginal and internal adaptation of endocrowns.

## Methods

**Fig. 1:**
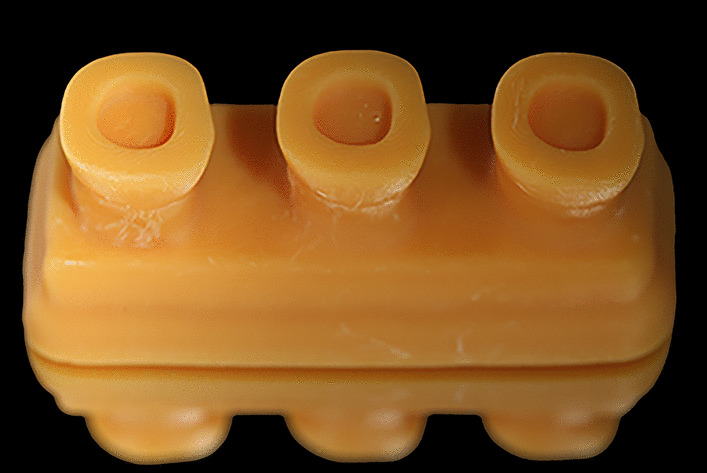
Standardized identical dies of endocrown prepared mandibular molar fabricated by using a 3D-printing system

A typodont mandibular first molar (A20A-200; NISSIN Dental Products, Inc., Kyoto, Japan) was prepared for a flat butt joint margin endocrown with a 2-mm occlusal reduction and 2-mm pulp chamber extension with an internal taper of 8° of the axial walls [30, 31]. The endocrown preparation margin was located on the enamel. Then, using a 3D-printing system (DLP1080E, HAN’S LASER, Shenzhen, China) and resin material (T-MRD-521, HAN’S LASER, Shenzhen, China), the prepared molar was replicated into 40 resin blocks, each containing 3 copies (Fig. 1), 120 specimens were produced.

**Fig. 2:**
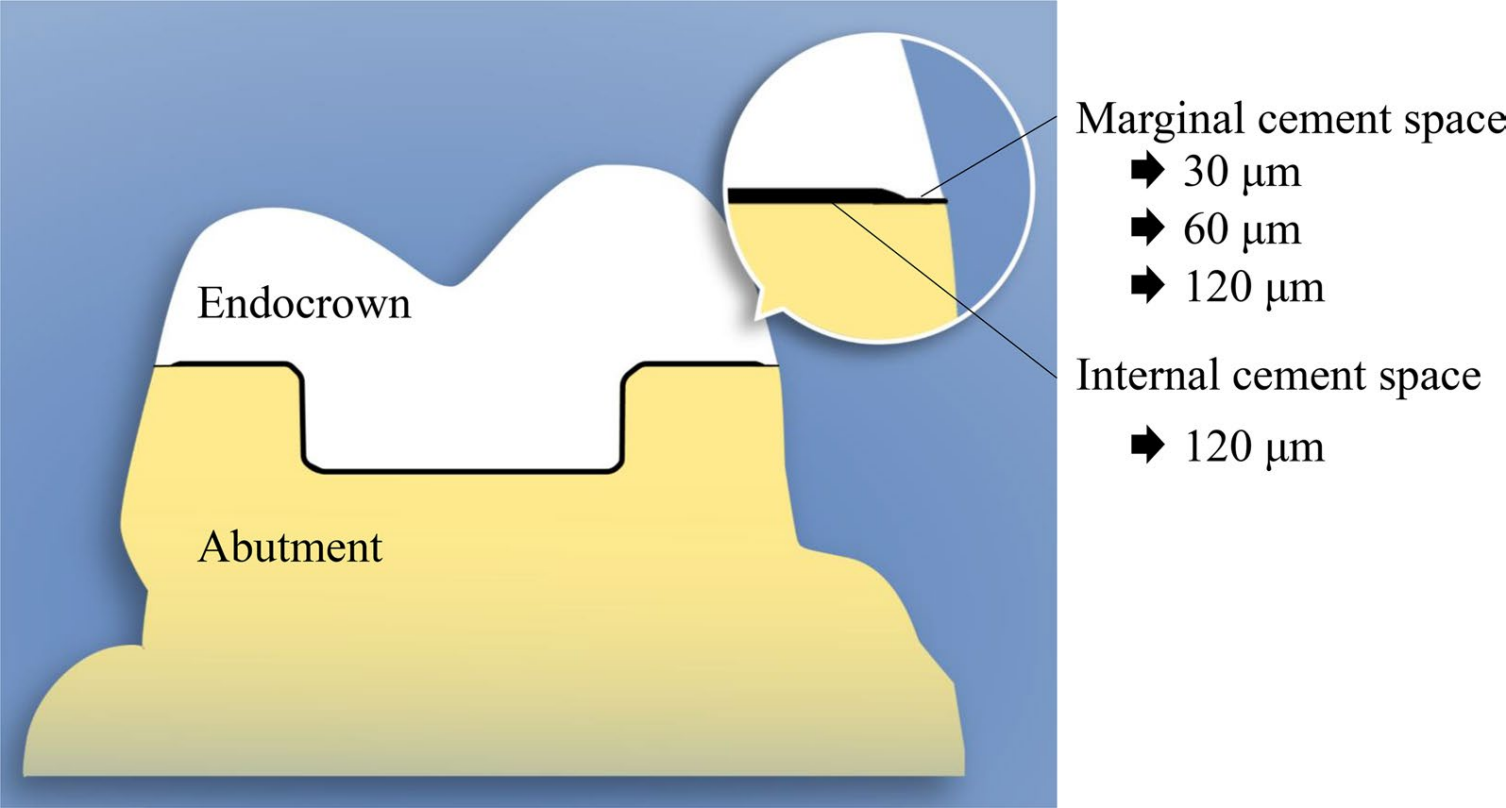
The cement space settings used in each experimental group

For all specimens, digital optical impressions were performed with an intraoral scanner, and thus 120 standard tessellation language (STL) files were acquired and imported into the CAD software (CEREC AC, Dentsply Sirona, York, PA, USA), ready to design the endocrown. According to the subsequent settings of the virtual CS (30, 60, and 120 μm), the STL files were randomly distributed into three groups (n = 40). The design parameters of the marginal adhesive gap were set to 30, 60, or 120 μm, in the CAD software (Fig. 2). All endocrowns were designed with identical external contours. In the milling process, using CAM software, these three groups were further divided into subgroups based on different CAD-CAM materials: Vita Suprinity (VS; VITA Zahnfabrik), Celtra Duo (CD; Dentsply Sirona), Lava Ultimate (LU; 3 M ESPE), and Grandio blocs (GR; VOCO). The characteristic features of the tested materials are listed in Table 1 [25, 32–34]. Ten endocrowns of each material were fabricated for each STL file with a specific CS setting.

**Table 1:** Characteristic features of materials tested

Material	Manufacturer	Class	Modulus of elasticity (GPa)	Flexural strength (MPa)	Hardness (MPa)
Vita Suprinity (VS)	Vita Zahnfabrik	Pre-sintered zirconia-reinforced lithium silicate glass-ceramic	104.9	420	558.1
Celtra Duo (CD)	Dentsply Sirona	Fully-sintered zirconia-reinforced lithium silicate glass-ceramic	107.9	370	463.5
Lava Ultimate (LU)	3 M ESPE	Resin composite	12.7	248.4	102.3
Grandio Blocs (GR)	VOCO	Resin composite	18.0	333	154.6

The stabilization of the fabricated endocrown to the corresponding models was performed using a siloxane material (GC Fit Checker Advanced, GC Dental Industrial Corp, Tokyo, Japan) under a constant load of 9.8 N for 10 min [35]. Thereafter, the marginal and internal gaps of all specimens were analyzed using high-resolution micro-computed tomography (micro-CT) images (ZKKS-MCT-Sharp, Zhongke Kaisheng Medical Technology Company, Guangzhou, China). Each sample in the scanning tube was placed perpendicular to the X-ray beam for scanning, by setting the following scanning parameters [29]: accelerating voltage of 70 kV, current of 100 μA, exposure time of 79 ms per frame, Al + Cu filter, and rotation step of 0.6° for a 180° rotation. The pixel size of each image was 15 μm. Approximately 1200 cross-sections were obtained from each sample.

**Fig. 3:**
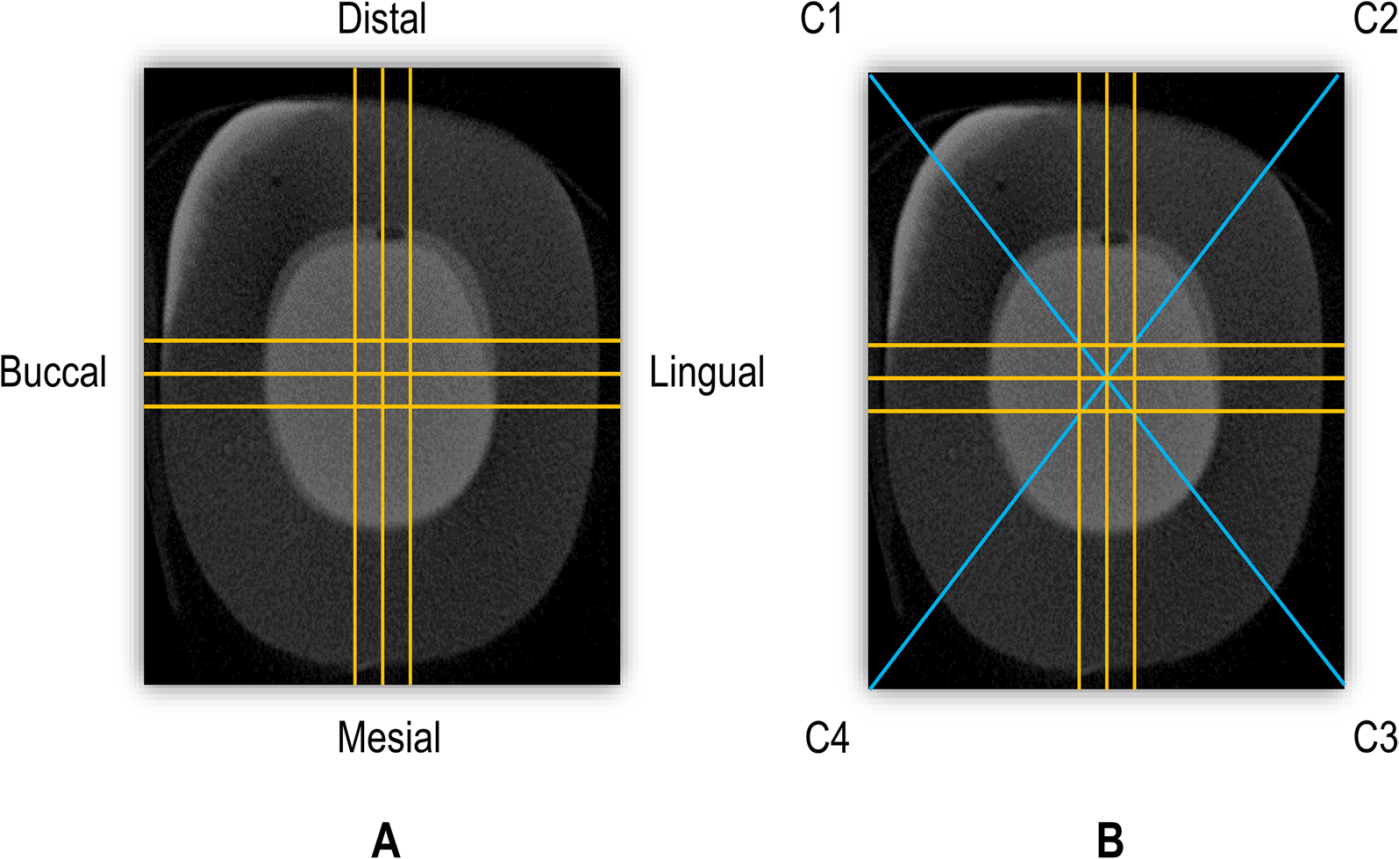
Micro-CT scan image (horizontal cut). **A** Selected sections in buccolingual and mesiodistal directions for internal and marginal adaptation. **B** Additional sections (C1-C4) for marginal adaptation

**Fig. 4:**
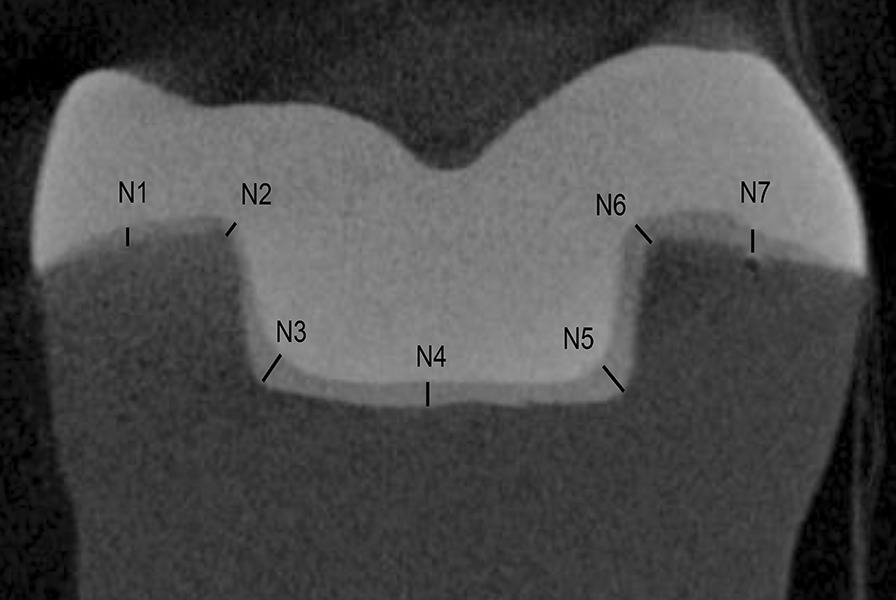
Micro-CT scan image (vertical sections). Schematic representation of measurement positions for internal adaptation. N1, N2, N6, and N7 were measured as the cervical seat, and N3, N4, and N5 were measured as pulpal floor

After loading the acquired projection images, the software (NRecon v1.6.9, Bruker Micro-CT, Billerica, MA, USA) automatically assimilated the images into a 3D reconstruction. Three vertical sections were selected from the core region of each specimen in the buccolingual (BL) and mesiodistal (MD) directions (Fig. 3A). Seven points (N1-N7) were selected on each of the BL and MD sections (Fig. 4) to analyze the internal fit. N1, N2, N6, and N7 were measured on the cervical seat, and N3, N4, and N5 were measured on the pulpal floor. A total of 42 measurements were taken for each specimen to analyze the internal fit. The same cross-sections used in internal adaptation were used to measure marginal fitness. In addition, two cross sections connecting the endocrown corners were added. A total of 16 points (Fig. 3B), including three points on each of the four sides, buccal (B1, B2, and B3), lingual (L1, L2, and L3), mesial (M1, M2, and M3), distal (D1, D2, and D3), and one point in each of the four corners (C1, C2, C3, and C4) of each specimen, were selected for marginal adaptation measurements [36]. The gap between the restoration and tooth at the selected points was measured in micrometers (µm).

Statistical analyses were performed using IBM SPSS Statistics, v23 (IBM Corp, Armonk, NY, USA). The normality assumption of the data and the homogeneity of the variances were checked using the Shapiro–Wilk and Levene’s tests, respectively. Because the data were normally distributed, 2-way analysis of variance (ANOVA) and Tukey’s honestly significant difference test were used (α = 0.05).

## Results

Regarding the variable CS settings, data analysis showed a statistically significant effect of CS settings on the marginal and internal discrepancy values of tested endocrowns (*p* < 0.001). For the marginal discrepancy, the highest values were recorded in group CS-30 (144.68–174.36 μm), followed by group CS-120 (81.74–105.35 μm), while the lowest values were recorded in group CS-60 (52.37–82.73 μm). Group CS-60 was statistically significantly different from the CS-30 or CS-120 groups (*p* < 0.001) (Table 2). All measured margin gap values were within the clinically acceptable range for CAD-CAM restorations, except for group CS-30. For the internal gap (Table 3), the CS-30 group showed significantly higher values (274.48–307.91 μm), in which all measurements exceeded the maximum clinically accepted value of 200 μm. When the CS was designed to be 60 or 120 μm, the internal gap values were within the acceptable range, and no statistically significant difference was found between these two groups (*p* > 0.05).

**Table 2:** Marginal discrepancy values of CAD-CAM endocrowns fabricated with four materials and three virtual cement spaces

Material	Cement space (µm)		
	30 (Mean ± SD)	60 (Mean ± SD)	120 (Mean ± SD)
VS	144.68 ± 22.43^bA^	52.37 ± 6.61^bC^	82.21 ± 10.85^bB^
CD	150.13 ± 19.03^bA^	59.89 ± 7.89^bC^	81.74 ± 14.57^bB^
LU	174.36 ± 22.78^aA^	82.73 ± 18.03^aC^	105.35 ± 13.59^aB^
GR	170.43 ± 26.04^aA^	79.22 ± 11.49^aC^	103.55 ± 7.82^aB^

Different superscript lowercase letters in each column indicate statistically significant differences between groups (*p* < 0.05). Different superscript uppercase letters in the same row indicate statistically significant differences (*p* < 0.05)

*VS* Vita Suprinity, *CD* Celtra Duo, *LU* Lava Ultimate, *GR* Grandio Blocs

**Table 3:** Internal discrepancy values of CAD-CAM endocrowns fabricated with four materials and three virtual cement spaces

Material	Cement space (μm)		
	30 (Mean ± SD)	60 (Mean ± SD)	120 (Mean ± SD)
VS	274.48 ± 82.06^aA^	121.11 ± 47.92^aB^	123.67 ± 44.05^aB^
CD	296.78 ± 82.92^aA^	123.61 ± 49.01^aB^	130.09 ± 45.18^aB^
LU	307.91 ± 77.44^aA^	128.00 ± 43.00^aB^	142.06 ± 35.64^aB^
GR	302.57 ± 88.03^aA^	129.64 ± 40.03^aB^	132.24 ± 38.56^aB^

Different superscript lowercase letters in each column indicate statistically significant differences between groups (*p* < 0.05). Different superscript uppercase letters in the same row indicate statistically significant differences (*p* < 0.05)

*VS* Vita Suprinity, *CD* Celtra Duo, *LU* Lava Ultimate, *GR* Grandio Blocs

Regarding the variable materials, all the tested groups displayed statistically significant differences in marginal fit (*p* < 0.001). Group VS and CD had a narrower marginal gap than the LU and GR groups (*p* < 0.001) (Table 2). No statistically significant differences were observed between the LU and GR groups or the VS and CD groups (*p* > 0.05). For the internal adaptation, no significant differences were observed between the four materials (*p* > 0.05) (Table 3).

To determine the effect of regions on the internal adaptations, the measurements of the internal gap were compared between the pulpal floor and cervical seat. In the space for all groups, the pulpal floor showed a significantly higher value than the cervical region (*p* < 0.05) (Table 4).

**Table 4:** Regional internal adaptation values: CAD-CAM endocrowns fabricated with four materials and three cement spaces

Material	Cement space (μm)	Region	
		Cervical seat (Mean ± SD)	Pupal floor (Mean ± SD)
VS	30	210.66 ± 23.53^B^	338.30 ± 68.61^A^
	60	81.36 ± 13.88^B^	160.86 ± 34.67^A^
	120	86.60 ± 11.08^B^	160.74 ± 31.22^A^
CD	30	242.05 ± 22.56^B^	351.50 ± 85.40^A^
	60	82.77 ± 12.95^B^	164.46 ± 35.47^A^
	120	98.43 ± 20.72^B^	161.76 ± 40.63^A^
LU	30	257.33 ± 22.87^B^	358.48 ± 79.95^A^
	60	99.83 ± 12.03^B^	156.18 ± 44.46^A^
	120	120.92 ± 14.33^B^	163.21 ± 38.16^A^
GR	30	230.41 ± 34.88^B^	374.73 ± 61.25^A^
	60	103.71 ± 12.04^B^	155.57 ± 41.55^A^
	120	106.71 ± 13.93^B^	157.77 ± 38.52^A^

Different superscript uppercase letters in each column indicate statistically significant differences between groups (*p* < 0.05)

*VS* Vita Suprinity, *CD* Celtra Duo, *LU* Lava Ultimate, *GR* Grandio Blocs

## Discussion

Ensuring that the gap between endocrowns and their abutments is within acceptable marginal and internal values is critical for a positive long-term outcome. This study evaluated the marginal and internal fit of CAD-CAM endocrowns fabricated using four materials (VS, CD, LU, and GR) and three CS settings (30, 60, and 120 μm). The results showed that differences in the CS settings significantly affected the marginal and internal adaptation of the endocrown, while the types of restorative materials only affected the marginal adaptation but not the internal fit. Therefore, the null hypothesis was partially rejected.

Adaptation can be achieved using various methods, including micro-CT imaging. It can provide accurate, 2D, or 3D high-quality images to show the restoration in any direction and orientation without invasion or destruction of the subject being investigated [4, 22, 37, 38]. In addition, precise visualization of the entire cement layer, as well as segmentation and quantification of different areas and materials, can be achieved [37]. Moreover, the selection of measurement points is crucial. Studies have recommended that at least 10 points be randomly selected to measure the adaptation of the restoration and that measurements should be performed throughout the restoration [36, 39]. In addition to the conventional margin measurement points in the six sections used in the previous study, the present study used another two cross-sections joining the endocrown corners for measurement, which enabled the higher reliability of our results.

The CAD-CAM fabricated endocrown has shown significant advantages in clinical practice, but some basic information still needs to be clarified through further investigations, including the optimal CS setting. Theoretically, there is a dilemma in CS designation. A narrow CS can achieve the best fitness of the fabricated restoration and abutment, but such a luting space would be difficult to manage in practice. A wide CS provides more luting space but might cause more microleakage and restoration detachment [15]. The lowest CS value of the CAD-CAM crown was suggested to be set at 50 μm, of which 30 μm was for the space of cement and avoidance of friction, and the remaining 20 μm was for possible deterioration during production [6, 22, 40]. However, discrepancies in placement were not considered in this study. In addition, this proposal has not been supported by experiments or clinical trials.

In this study, three scales of CS values were chosen based on previous reports [4, 6, 7, 21], in which 30 and 120 μm were the lowest and highest acceptable values, respectively; 60 μm was the median of 30 and 120, and, arguably, 60 μm was slightly larger than the suggested 50 μm. Unexpectedly, the CS-30 group showed the highest mean gap width, while the CS-60 group generated the smallest marginal and internal gaps. The relationship between the CS setting in software and the marginal discrepancy in restored teeth has recently attracted attention, and previous reports mentioned that the marginal gap increased with a decrease in the CS setting values [18, 21]. A smaller CS prevents the restoration of complete seating, thus exacerbating the marginal gap. In addition, a negative correlation between the CS setting value and the time for manual crown adjustment was reported [41], indicating that a larger CS value is a possible strategy to improve adaptation and reduce internal adjustments. However, when the CS was designed to be even wider, reaching the maximum threshold recommended by the manufacturer (120 μm), the marginal fit of the restorations worsened, although the actual gap width measurements were still within the acceptable range. The discrepancy between the designation and reality may be due to errors in restoration production, placement inaccuracies, and difficulties in expressing excess cement.

Due to the variable elasticity modulus and milling properties, different CS designations may be preferred to improve CAD-CAM restoration fitness when utilizing different materials [23, 25, 26]. Four products composed of ceramic materials (VS and CD) or resin composites (LU and GR) were used in this study. The possibility of a role for materials in adaptation was verified in the present study. When the CS was set to 60 μm in the software, the VS and CD materials generated an actual marginal gap width close to the predesigned 60 μm, which was better than that of the LU and GR materials. When the CS was set at 120 μm, the actual width of the gap was close to 120 μm in the restorations made of LU and GR. Ceramics have a higher modulus of elasticity and can be more accurately milled and hardly deformable during production [7, 26]. On the contrary, resin composites have better machinability and adaptation [4, 29]; therefore, less precision in milling and more deformation might occur during late processing. In practice, a high agreement between the designation and reality is always preferred. Our results indicate that the settings of CS must be changed according to the material used.

In this study, the effect of crystallization on the fit of VS versus CD endocrowns was investigated. Compared with the fully crystallized ceramic block (CD), VS is a partially crystallized ceramic block that requires post-milling crystallization to achieve its maximum mechanical and optical properties. During the crystallization process, the lithium silicate and meta-silicate crystals become smaller, and densification increases with a reduction in particle size for a close-packed arrangement [42]. Previous studies reported that densification shrinkage during the crystallization process affects the adaptation of ZLS crowns [8, 24]. However, our study found no significant differences between VS and CD. This difference can be attributed to the preparation design. The risk of causing a marginal discrepancy is lower in endocrowns with a flat butt upper gingival shoulder, as used in this study, than that in other restoration designs. Therefore, post-sintering shrinkage of the ZLS material did not significantly affect the maintenance of a “good fit”.

To analyze the internal fit in more detail, we divided the measurements of the internal gap into two different areas for accurate comparisons: the cervical seat and the pulpal floor (Table 4). Consistent with the present study, the internal gap in the pulp chamber has been reported to be the worst-fitting area [4, 7, 36]. This may be due to the narrow and complex structure of the pulpal chamber, and the limited optical depth of the scanner, resulting in blurred images of the pulpal area [4, 7, 36, 43].

This study focused on the influence of CS setting and restorative material factors on the adaptation of a CAD-CAM endocrown. The results showed that a CS setting of 30 μm would generate poor fitness, while a setting of 60 μm or 120 μm would be acceptable. Moreover, considering the need for a strong correlation between design and reality, a space setting of 60 μm would be better for ceramic materials, and 120 μm would be better for resin composites. Further detailed experimental studies and clinical trials are required in the future. To the best of our knowledge, this is the first relevant study on CS designation according to different materials.

Our study has several limitations. As an in vitro experiment, this could not include in vivo factors, such as patient cooperation and contamination with saliva and blood during scanning processing. In addition, a single-tooth model and one scanning and milling system during the design and fabrication of CAD-CAM endocrowns eliminate inconsistencies but overlook diversity in the real world [9, 44]. Further studies are required to understand the influence of clinical oral conditions and the diversity of the CAD-CAM system on the adaptation of endocrowns.

## Conclusion

Within the limitations of this in vitro study, the following conclusions were drawn:

1. The setting of virtual CS had a significant effect on the adaptation of CAD-CAM endocrown restoration. Setting at 30 μm would generate poor fitness, while 60 μm or 120 μm would be acceptable.
2. When considering the high coincidence between design and reality, a space setting at 60 μm would be better for ceramic material and at 120 μm would be better for resin composite.

## Data Availability

All data generated or analyzed during the current study are available from the corresponding author on reasonable request.

## Abbreviations

CS: Cement space
VS: Vita suprinity
CD: Celtra duo
LU: Lava ultimate
GR: Grandio blocs
CAD-CAM: Computer-aided design and computer-aided manufacturing
3D: 3-Dimensional
STL: Standard tessellation language
BL: Buccolingual
MD: Mesiodistal
